# A susceptibility biomarker identification strategy based on significantly differentially expressed ceRNA triplets for ischemic cardiomyopathy

**DOI:** 10.1042/BSR20191731

**Published:** 2020-01-24

**Authors:** Yuqing Zou, Yahui Wang, Zherou Rong, Benliang Wei, Yang Liu, Zhaona Song, Wenshuai Li, Erqiang Hu, Gui Deng, Yuehan He, Junjie Lv, Lina Chen, Wan Li

**Affiliations:** College of Bioinformatics Science and Technology, Harbin Medical University, Harbin, Heilongjiang Province, China

**Keywords:** ceRNA, ICM, lncRNA, miRNA, mRNA

## Abstract

Ischemic cardiomyopathy (ICM) is a common human heart disease that causes death. No effective biomarkers for ICM could be found in existing databases, which is detrimental to the in-depth study of this disease. In the present study, ICM susceptibility biomarkers were identified using a proposed strategy based on RNA-Seq and miRNA-Seq data of ICM and normal samples. Significantly differentially expressed competing endogenous RNA (ceRNA) triplets were constructed using permutation tests and differentially expressed mRNAs, miRNAs and lncRNAs. Candidate ICM susceptible genes were screened out as differentially expressed genes in significantly differentially expressed ceRNA triplets enriched in ICM-related functional classes. Finally, eight ICM susceptibility genes and their significantly correlated lncRNAs with high classification accuracy were identified as ICM susceptibility biomarkers. These biomarkers would contribute to the diagnosis and treatment of ICM. The proposed strategy could be extended to other complex diseases without disease biomarkers in public databases.

## Introduction

Ischemic cardiomyopathy (ICM) is a common heart disease and a major cause of heart failure as well as sudden cardiac death with the pathological characteristics of myocardial ischemia and fibrous tissue hyperplasia. Pathogenic factors for ICM include genetics, metabolic defect, inflammation, coronary artery lesion and so on [1]. Increasing evidence exhibited that individualized gene test could better identify individuals with high ICM risk in order to have early treatment prior to symptom onset [2]. Gene therapy can be used to target ICM biomarkers to change the myocardium microenvironment and improve heart function. However, only few ICM biomarkers were found in studies, and none are stored in public databases. Thus filling the gap that ICM biomarkers are rarely identified at present is of importance for early diagnosis and treatment of ICM patients. One of the major challenges in filling this gap is the cost of experimental approaches in terms of time and labor. Therefore, it is necessary to identify more effective ICM biomarkers using computational biology strategies.

As the development and popularization of next-generation sequencing technologies, disease-associated genes or mutations have been identified effectively from lots of sequencing data [3]. Additionally, it was expected that non-coding RNAs would become promising therapeutic targets for cardiovascular diseases [4,5]. Over the past several years, increasing lines of evidence suggested that competing endogenous RNAs (ceRNAs) emerged as an important class of post-transcriptional regulator that altered gene expression through a miRNA-mediated RNA–RNA interaction mechanism [6–8]. Expression of mRNAs, miRNAs, and lncRNAs could also be obtained from sequencing data, alternation of which could influence the function and metabolism of cells through ceRNA triplets. With the development of bioinformatics technology, data analysis or mining methods have been applied to studies of ceRNA triplets or networks [9]. For example, lncRNAs acted as ceRNAs regulating genes were found to be involved in left ventricular systolic function [10]. In addition, regulatory role of ceRNA cross-talk with cancer-associated genes has been reported in the progression of various types of cancer [11–13].

**Figure 1:**
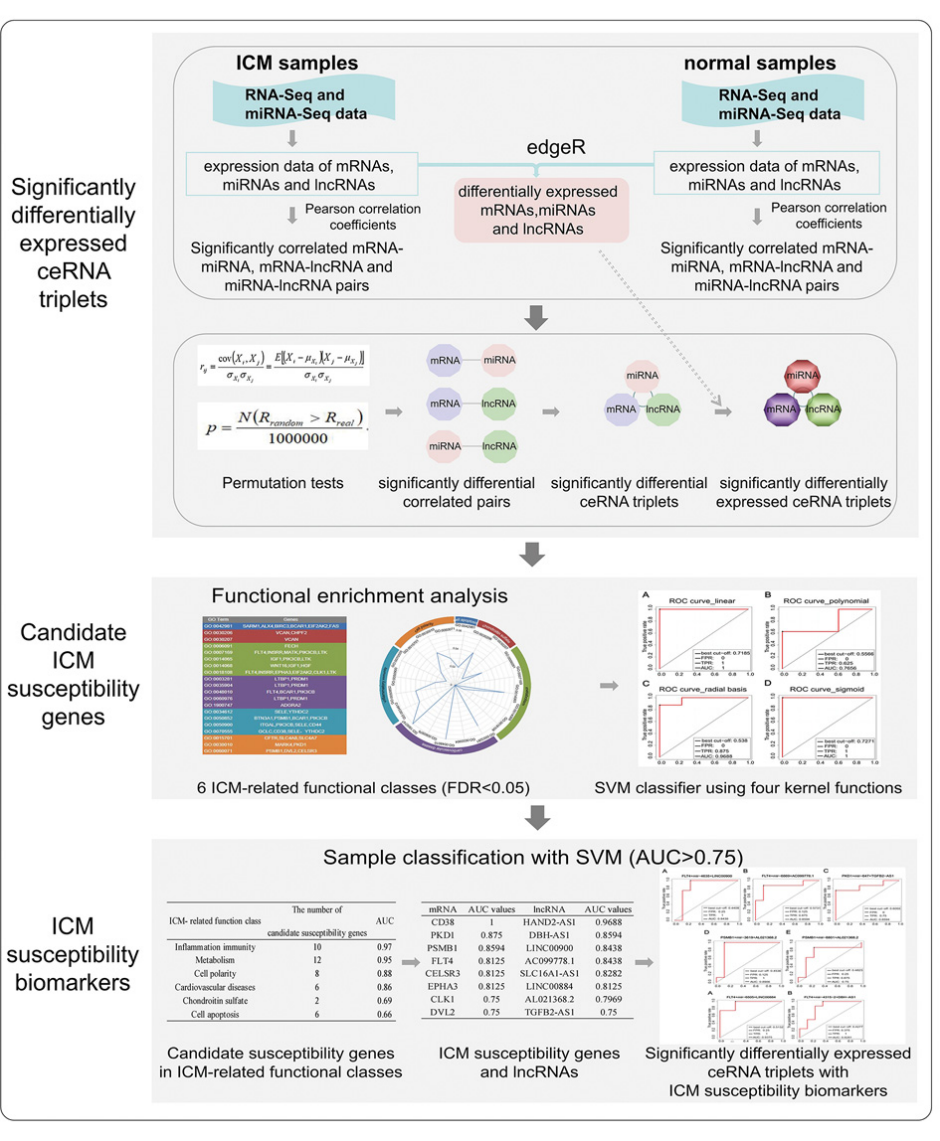
A schematic representation of the ICM biomarker identification strategy adopted in the present study

In the present paper, ICM susceptibility biomarkers were identified using a proposed strategy from expression, functions and regulation relationships of mRNAs, miRNAs, and lncRNAs based on RNA-Seq and miRNA-Seq data of ICM and normal samples. First, significantly differentially expressed ceRNA triplets composed of significantly differential correlated pairs among mRNAs, miRNAs, and lncRNAs were constructed. Then, functional enrichment analysis was conducted for differentially expressed genes in these significantly differentially expressed ceRNA triplets to select candidate ICM susceptibility genes. Finally, ICM susceptibility biomarkers, including ICM susceptibility mRNAs, miRNAs, and lncRNAs, were further identified based on classification performance (Figure 1).

## Materials and methods

### Data

The RNA-Seq and miRNA-Seq data (GSE46224) based on the platform of GPL11154 were downloaded from the Gene Expression Omnibus database (https://www.ncbi.nlm.nih.gov/geo/) [14], in which eight ICM and eight normal samples were used for ICM biomarker identification. Reference human genome (hg38.9) and genome annotation information were downloaded from Ensemble (ftp://ftp.ensembl.org/pub/release-91/) [15].

RNA-Seq data were processed using the following pipeline. Fastq files were converted with the SRA ToolKit for paired-end sequencing runs. Sequencing reads were mapped to reference human genome (hg38.9) using HISAT2. Then, sequence alignment/map (.SAM) format files were converted into binary alignment/map (.BAM) format using SAMTools [16]. StringTie was used for transcript assembling [17]. Counts for mRNAs and lncRNAs were obtained by htseq-count [18]. For miRNA-Seq data, the miRDeep2 software package was employed to mine miRNAs and the quantifier module in this package for miRNA read counts [19]. The read counts were further normalized into fragments per kilobase of transcript per million (FPKM) values for mRNAs, miRNAs, and lncRNAs by Ballgown and then compiled into a matrix [20]. The expression data of 18281 mRNAs, 1049 miRNAs and 8996 lncRNAs for every sample were extracted after the preprocessing step.

Between ICM and normal samples, 4736 mRNAs, 21 miRNAs, and 952 lncRNAs were identified to be differentially expressed by edgeR [21] with *P*<0.05 using read counts as input.

### Construction of significantly differentially expressed ceRNA triplets

Correlation relationships between mRNAs, miRNAs and lncRNAs were used to construct significantly differentially expressed ceRNA triplets. Pearson correlation coefficients were employed to evaluate the relationships, which were calculated for pairs among mRNAs, miRNAs, and lncRNAs for ICM and normal samples, respectively (eqn 1).

(1) $$r_{ij}=\frac{\mathrm{cov}\left(X_{i},X_{j}\right)}{\sigma_{X_{i}}\sigma_{X_{j}}}=\frac{E\left\lfloor \left(X_{i}-\mu_{X_{i}}\right)\left(X_{j}-\mu_{X_{j}}\right)\right\rfloor }{\sigma_{X_{i}}\sigma_{X_{j}}}$$

where *X_i_* and X*_j_* are expression values for one mRNA, miRNA, or lncRNA *i* and another mRNA, miRNA, or lncRNA *j*. $\sigma_{X_{i}}$ and $\sigma_{X_{j}}$ are standard deviations, and $\mu_{X_{i}}$ as well as $\mu_{X_{j}}$ are mean values for *X_i_* and *X_j_*, respectively. cov(*X_i_,X_j_*) is the covariance between *X_i_* and *X_j_*. *E* represents expectation.

Significantly correlated mRNA–miRNA, mRNA–lncRNA, and miRNA–lncRNA pairs were screened out by the significantly negative correlation between miRNAs and mRNAs or lncRNAs, and positive correlations between mRNAs and lncRNAs (*P*<0.05).

In the present study, lncRNA–miRNA and mRNA–miRNA interactions were further determined according to mirCode (http://www.mircode.org).

Permutation tests were conducted to screen out significantly differential correlated pairs. Differences of Pearson correlation coefficients of mRNA–miRNA, mRNA–lncRNA, or miRNA–lncRNA pairs between ICM and normal samples were calculated and compared with 1000000 random selected differences, respectively. The statistical significance was determined by *P*-value, which was calculated as follows:

(2) $$P=\frac{N\left(R_{\text{random}}>R_{\text{real}}\right)}{1000000}$$

where *R*_real_ represents the real difference of Pearson correlation coefficients between ICM and normal samples, *R*_random_ represents a random one, and *N*(*R*_random_ > *R*_real_) represents the frequency of the random difference larger than the real one. Significantly correlated pairs with FDR-adjusted *P*-values < 0.05 were considered as significantly differential correlated pairs.

The mRNAs, lncRNAs, and their common significantly correlated miRNAs could form ceRNA triplets (mRNA–miRNA–lncRNA). Then from these significantly differential correlated pairs, significantly differential ceRNA triplets were built. Taking expression of mRNAs, miRNAs, and lncRNAs into consideration, significantly differential ceRNA triplets containing differentially expressed mRNAs, miRNAs, or lncRNAs were selected as significantly differentially expressed ceRNA triplets.

### Candidate ICM susceptibility genes

For complex diseases, the disease-related genes were expected to be from differentially expressed genes [22]. Thus, candidate ICM susceptibility genes were selected from differentially expressed genes.

For differentially expressed genes in significantly differentially expressed ceRNA triplets containing at least one differentially expressed miRNA or lncRNA, the gene ontology (GO) enrichment analyses using biological process (BP) were conducted to identify the significantly overrepresented GO terms using hypergeometric tests (eqn 3):

(3) $$P=1-\sum_{i=0}^{k-1}\frac{\left(\begin{array}{c} M\\ k \end{array}\right)\left(\begin{array}{c} N-M\\ n-k \end{array}\right)}{\left(\begin{array}{c} N\\ n \end{array}\right)}$$

where *N* is the number of genes in all functions, *n* is the number of differentially expressed genes in significantly expression differential triplets including at least one differentially expressed miRNA or lncRNA, *M* is the number of genes in a specific function, and *k* is the number of overlapping genes. The FDR-adjusted *P-*value < 0.05 was set as the criterion.

Differentially expressed genes enriched in ICM-related functional classes were regarded as candidate ICM susceptibility genes.

### Identification of ICM susceptibility biomarkers

Classifiers are frequently constructed for predicting samples of various statuses [23,24] to assess the reliability of identified genes [25,26]. Here, support vector machine (SVM) classifiers based on four kernel functions (linear, sigmoid, polynomial, radial basis) with gene expression values as classification features was constructed to distinguish ICM and normal samples. The performance of the classifiers was evaluated by the leave-one-out cross-validation method. The classification accuracy was assessed by the area under the receiver operating characteristic curve (AUC), which was a popular metric widely used. The genes with individual and joint classification accuracy higher than 0.75 were identified as ICM susceptibility genes. ICM susceptibility miRNAs or lncRNAs were identified in the same way. These ICM susceptibility genes, miRNAs and lncRNAs were ICM susceptibility biomarkers.

## Results

### Significantly differentially expressed ceRNA triplets

Significantly differential correlated pairs between ICM and normal samples were screened out with permutation tests (FDR < 0.05), which contained 674850 mRNA–miRNA pairs, 303790 miRNA–lncRNA pairs, and 7416284 mRNA–lncRNA pairs. Based on these significantly differential correlated pairs, significantly differential ceRNA triplets were built, of which 6464 were selected from disease samples and 7738 from normal samples.

Significantly differentially expressed ceRNA triplets were selected from significantly differential ceRNA triplets as those with differentially expressed mRNAs, miRNAs, or lncRNAs (Table 1). Only a few differentially expressed miRNAs and a large number of differentially expressed mRNAs and lncRNAs were contained in significantly differentially expressed ceRNA triplets. More significantly differentially expressed ceRNA triplets with two or three differentially expressed mRNAs, miRNAs, or lncRNAs were constructed from the ICM samples.

**Table 1:** The number of significantly differentially expressed ceRNA triplets and differentially expressed elements

	Significantly differentially expressed ceRNA triplets	Differentially expressed mRNA	Differentially expressed miRNA	Differentially expressed lncRNA
NT1	2643	178	9	227
T1	2112	151	9	0
NT2	275	115	6	153
T2	507	101	8	154
NT3	1	1	1	1
T3	25	13	3	14
Total	5563	240	17	363

NT1, NT2, and NT3 represent significantly differentially expressed ceRNA triplets with one, two, and three differentially expressed mRNAs, miRNAs, or lncRNAs of the normal samples, respectively; T1, T2, T3 represent those of the ICM samples, respectively.

### Candidate ICM susceptibility genes

**Figure 2:**
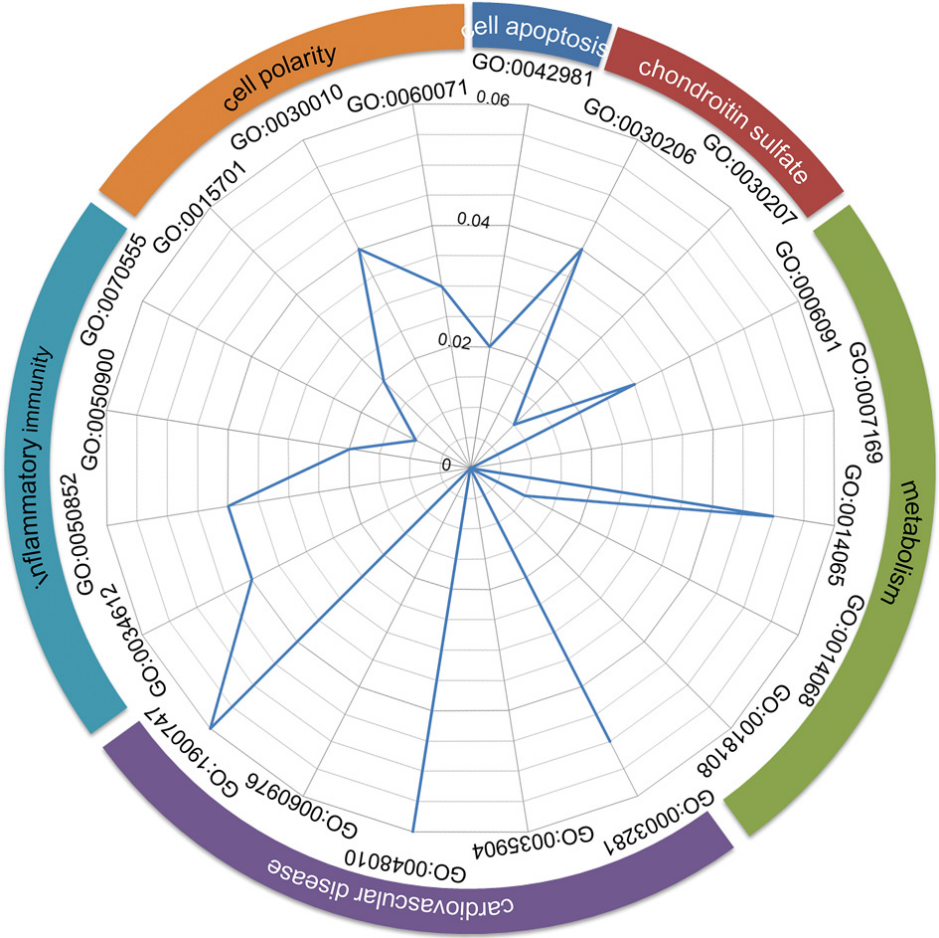
Functional enrichment analysis of differentially expressed genes in significantly differentially expressed ceRNA triplets ICM-related functional classes (the outer ring) and GO terms (the inner ring) significantly enriched by differentially expressed genes in significantly differentially expressed ceRNA triplets including at least one differentially expressed miRNA or lncRNA. The axis indicates FDR-adjusted *P*-value of the enrichment analyses.

To screen out candidate susceptibility genes, differentially expressed genes in significantly differentially expressed ceRNA triplets including at least one differentially expressed miRNA or lncRNA were used for further screening. Functional enrichment analysis was carried out to 68 differentially expressed genes in these triplets. Thirty-seven GO terms were significantly enriched by these genes (FDR-adjusted *P*-value < 0.05), 20 of which were involved in 6 ICM-related functional classes, including cardiovascular disease, cell apoptosis, metabolism, inflammatory immunity, chondroitin sulfate, and cell polarity (Figure 2).

Due to ICM, ‘cardiovascular disease’ remains the leading cause of death among women globally [27]. Engrafted cardiac stem cells are subjected to acute ‘cell apoptosis’ in the ischemic microenvironment, attenuation of which suggested a new clue enhancing the survival rate in the infarcted myocardium for cell therapy in ICM [28]. Ko et al. found that myocardial glucose uptake could discriminate between viable and non-viable myocardium, and may be prognostic predictors of cardiovascular death in patients with ICM, after prospectively quantifying the rate of myocardial glucose uptake in myocardium with different perfusion-‘metabolism’ patterns [29]. ICM is in part an immune-mediated disease [30]. The immune system was reported to play a central role in ‘inflammatory immunity’ aimed at repairing ischemic myocardium [31]. Disruption of the ‘cell polarity’ complex could cause loss of polarized cardiomyocyte division and loss of normal myocardial trabeculation [32]. The oligosaccharide ‘chondroitin sulfate’ could promote the proliferation of normal myocardial cells [33].

**Figure 3:**
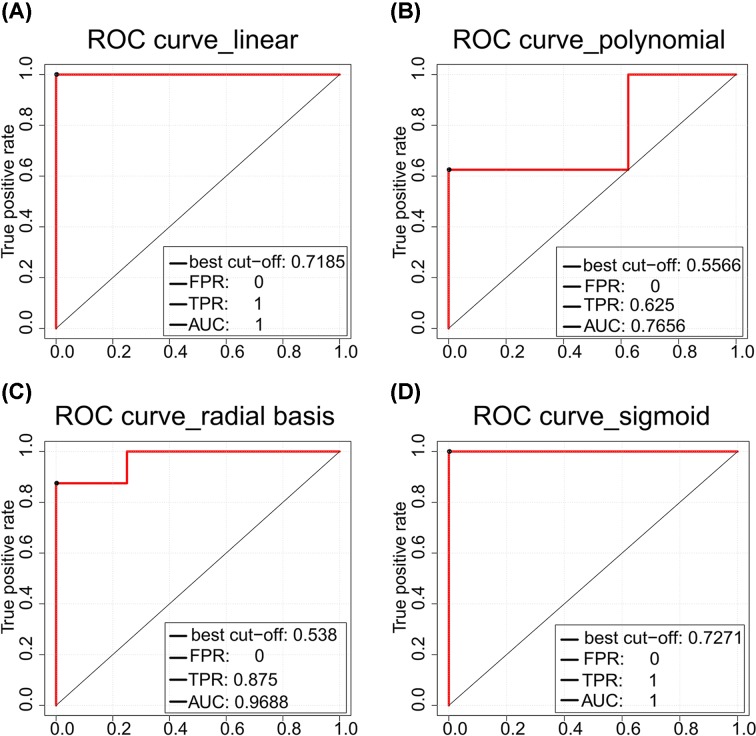
Classification accuracy of 37 candidate ICM susceptibility genes ROC curves of the SVM classifier with 37 candidate ICM susceptibility genes as classification features using four kernel functions: (**A**) the linear kernel, (**B**) the polynomial kernel, (**C**) the sigmoid kernel, and (**D**) the radial basis function kernel.

In these ICM-related functional classes, 37 differential genes were regarded as candidate ICM susceptibility genes. With expression values of these candidate ICM susceptibility genes as classification features, ICM and normal samples could be classified accurately based on the SVM classifier using four kernel functions. The SVM classifier using linear and sigmoid kernel functions had the best classification accuracy (Figure 3).

### ICM susceptibility biomarkers

ICM and normal samples were classified based on the SVM classifier using the linear kernel function with candidate ICM susceptibility genes in each ICM-related functional class as classification features (Table 2). It was showed that candidate ICM susceptibility genes in four ICM-related functional classes could classify samples of different statuses with high accuracy (AUC > 0.85).

**Table 2:** Classification efficiency of candidate susceptibility genes in ICM-related functional classes

ICM-related functional class	The number of candidate susceptibility genes	AUC
Inflammation immunity	10	0.97
Metabolism	12	0.95
Cell polarity	8	0.88
Cardiovascular diseases	6	0.86
Chondroitin sulfate	2	0.69
Cell apoptosis	6	0.66

Furthermore, each candidate ICM susceptibility gene in these four functional classes was used as a classification feature to classify samples with an SVM classifier, respectively. Eight genes with AUC values > 0.75 were identified to be ICM susceptibility genes (Table 3).

**Table 3:** Classification accuracy of ICM susceptibility genes and lncRNAs

mRNA	AUC values	lncRNA	AUC values
CD38	1	HAND2-AS1	0.9688
PKD1	0.875	DBH-AS1	0.8594
PSMB1	0.8594	LINC00900	0.8438
FLT4	0.8125	AC099778.1	0.8438
CELSR3	0.8125	SLC16A1-AS1	0.8282
EPHA3	0.8125	LINC00884	0.8125
CLK1	0.75	AL021368.2	0.7969
DVL2	0.75	TGFB2-AS1	0.75

Each lncRNA or miRNAs in significantly differentially expressed ceRNA triplets including ICM susceptibility genes was further used as a classification feature to classify samples with an SVM classifier, respectively. Eight lncRNAs with AUC values > 0.75 were identified to be ICM susceptibility lncRNAs (Table 3). miRNAs could not distinguish between disease and normal samples very well (AUC < 0.75). Therefore, no miRNAs were identified as ICM susceptibility miRNA.

In total, eight ICM susceptibility genes (CD38, PKD1, PSMB1, FLT4, CELSR3, EPHA3, CLK1, and DVL2) and eight ICM susceptibility lncRNAs (TGFB2-AS1, AL021368.2, LINC00884, LINC00900, AC099778.1, DBH-AS1, HAND2-AS1, and SLC16A1-AS1) were identified as ICM susceptibility biomarkers.

Similarly, SVM classifiers based on the other three kernel functions (sigmoid, polynomial, radial basis) with these ICM susceptibility genes and lncRNAs were also built. AUC values of susceptibility biomarkers were also higher than 0.75, suggesting high classification accuracy and stable classification efficiency of these susceptibility biomarkers.

**Figure 4:**
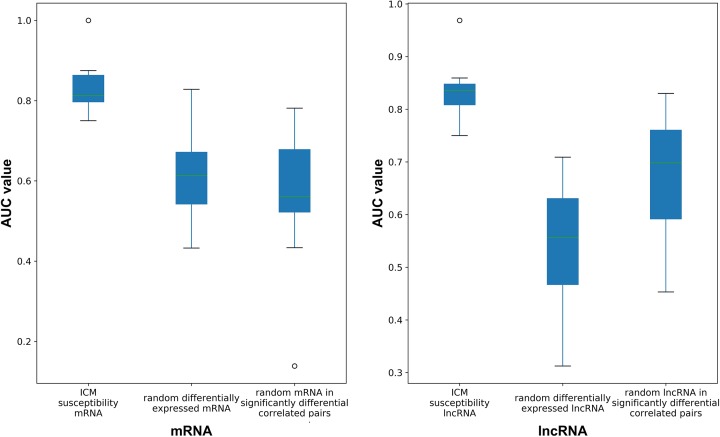
Classification accuracy comparison of ICM susceptibility biomarkers Classification accuracy of ICM susceptibility biomarkers (mRNAs and lncRNAs) and randomly selected mRNAs and lncRNAs from those differentially expressed or in significantly differential correlated pairs.

To further validate the disease correlation of ICM susceptibility biomarkers, the classification accuracy of these ICM susceptibility biomarkers (mRNAs and lncRNAs) was compared with the same number of randomly selected mRNAs and lncRNAs from those differentially expressed or in significantly differential correlated pairs, respectively. Both the ICM susceptibility genes and lncRNAs we identified had the highest classification accuracy (Figure 4). These results indicated that the ICM susceptibility biomarkers were more correlated with ICM than other mRNAs or lncRNAs.

No ICM susceptibility genes or lncRNAs were verified to be directly associated with ICM by literature. However, five ICM susceptibility genes and two ICM susceptibility lncRNAs were found to be involved in heart-related processes or functions. Activation of PKD1 was shown to be as obligatory for contraction-induced glucose transporter type-4 translocation in cardiac muscle, which was essential to stimulate cardiac glucose uptake during increased energy demand [34]. EPHA3 was associated with reduced bromodeoxyuridine incorporation in cardiomyocytes [35]. CD38 plays an essential role in cardiac hypertrophy since the cardiac hypertrophy was much more severe in wild-type mice compared with CD38 knockout mice. Thus, CD38 could be a novel target for treating cardiac hypertrophy [36]. The cell surface marker FLT4 specifically identify and enrich for a cardiovascular progenitor cell with trilineage cardiovascular potential *in vitro* and the robust ability for differentiation into mature adult cardiomyocytes *in vivo* [37]. DVL2, involved in outflow tract development, is a direct target of miR-138. Further, the functional variant rs139365823 in pre-miR-138 enhanced the miR-138-mediated inhibitory regulation of DVL2 and conferred the risk for congenital heart disease in a Chinese population [38]. LncRNA LINC00884 was one of the top five lncRNAs with the largest numbers of ICM associations in the study of He et al. [39].

Other three ICM susceptibility genes, CLK1, PSMB1, and CELSR3, were enriched in ICM-related functional classes, metabolism, inflammatory immunity, and cell polarity, respectively. And one ICM susceptibility lncRNA HAND2-AS1 was involved in energy metabolism, an ICM-related functional class since its knockdown promoted the expression level of a serious of enzymes that involved in energy metabolism [40]. Subsequently, these three ICM susceptibility genes and one ICM susceptibility lncRNA might participate in the pathogenesis and progression of ICM. Further validation of other lncRNAs is necessary to confirm their importance in the context of ICM.

## Discussion

ICM is a common heart disease that causes death in humans. No ICM biomarkers were deposited in existing disease associated databases. To identify ICM susceptibility biomarkers, in the present paper, a susceptibility biomarker identification strategy based on significantly differentially expressed ceRNA triplets was proposed. For ICM and normal samples, significantly differentially expressed ceRNA triplets composed of significantly differential correlated pairs among mRNAs, miRNAs, and lncRNAs were constructed. Differentially expressed genes in these significantly differentially expressed ceRNA triplets were enriched in ICM-related functional classes. Combining functional information, ICM susceptibility biomarkers, including eight ICM susceptibility mRNAs and eight ICM susceptibility lncRNAs, were further identified based on classification performance.

The ICM susceptibility genes enriched in four ICM-related functional classes could classify samples accurately, including cardiovascular disease, which was associated with coronary angiogenesis and vascular endothelial growth factor receptor [41]; inflammatory immune, which was capable of causing myocardial ischemic injury [42]; metabolism, which was related to the phosphorylation of PI3K and Akt in myocardial tissue of cardiomyopathy [43], and cell polarity, one of the fundamental causes of congenital heart disease [44].

**Figure 5:**
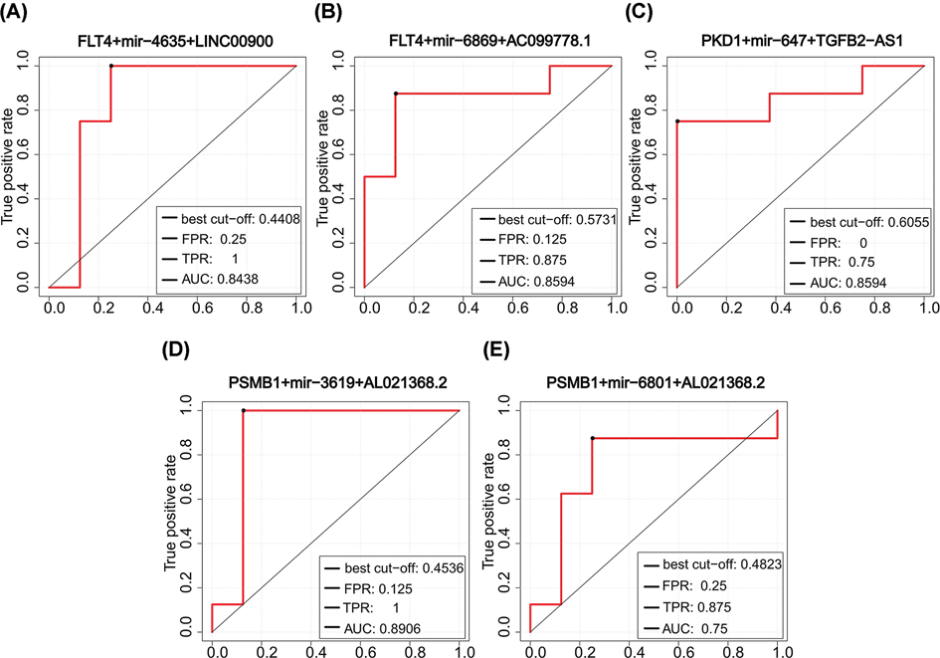
Classification performance of five significantly differentially expressed ceRNA triplets from the ICM samples ROC curves of significantly differentially expressed ceRNA triplets composed of (**A**) FLT4+mir-4635+LINC00900, (**B**) FLT4+mir-6869+AC099778.1, (**C**) PKD1+mir-674+TGFB2-AS1, (**D**) PSMB1+mir-3619+AL021368.2, and (**E**) PSMB1+mir-6801+AL021368.2.

**Figure 6:**
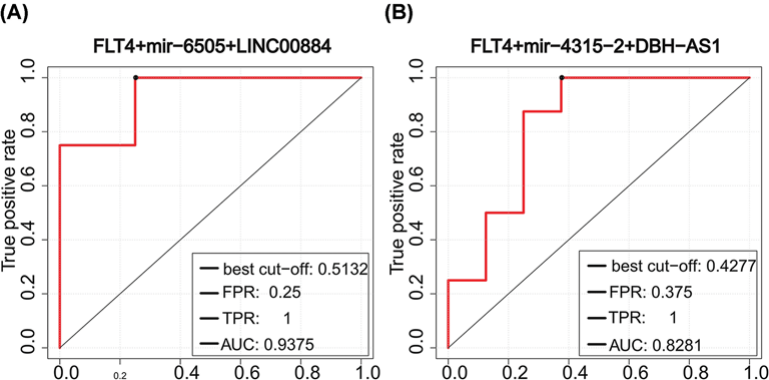
Classification performance of two significantly differentially expressed ceRNA triplets from the normal samples ROC curves of significantly differentially expressed ceRNA triplets composed of (**A**) FLT4+mir-6505+LINC00884 and (**B**) FLT4+mir-4315-2+DBH-AS1.

The ICM susceptibility biomarkers we identified had high classification accuracy (AUC > 0.75) and had the potential to be diagnostic markers. These susceptibility biomarkers formed 10 significantly differentially expressed ceRNA triplets, 7 of which were identified from the ICM samples, and 3 were from the normal samples. The classification efficiency of each of the 10 significantly differentially expressed ceRNA triplets was further evaluated. AUC values of seven triplets were higher than 0.75, of which five were from the ICM samples (Figure 5) and two from the normal samples (Figure 6). These seven triplets contained three ICM susceptibility genes (FLT4, PSMB1, and PKD1) and six susceptibility lncRNAs. It was worth highlighting that FLT4 was in four of these significantly differentially expressed ceRNA triplets, which was enriched in cardiovascular and metabolic functional classes and shown to be associated with the development of the heart aorta [45]. So changes that have taken place in this gene itself and its regulatory elements may disorder myocardial function. Other two significantly differentially expressed ceRNA triplets from the normal samples (Figure 6) showed similar and good classification accuracy while two differentially expressed miRNAs with poor classification accuracy were contained. This indicated that mRNAs and lncRNAs might play vital roles in the classification.

To exhibit the generalizability of our susceptibility biomarker identification strategy, it was performed on another complex disease, chronic obstructive pulmonary disease (COPD). The data (GSE57148) based on the platform of GPL11154 was downloaded from the GEO database, which contained 98 ICM and 91 normal samples. Eight COPD susceptibility genes (CBX5, MIDN, FAM136A, NUFIP2, HMGN2, MPLKIP, MICA, and RLIM) and seven COPD susceptibility lncRNAs (LINC00654, AC020978.8, AP006284.1, AC145207.5, NPTN-IT1, PSMD6-AS2, and ADAMTSL4-AS1) were identified as COPD susceptibility biomarkers. Classification accuracy of these COPD susceptibility biomarkers as classification features was evaluated. COPD susceptibility mRNAs (AUC = 0.827) or COPD susceptibility lncRNAs (AUC = 0.853) could distinguish between COPD and normal samples accurately. Moreover, more than half of these COPD susceptibility genes were validated to be associated with COPD in literature of COPD-related researches.

The present study has several limitations that need to be addressed in future studies. First, the sample size of the RNA-Seq data we used was relatively small. Second, RNA-Seq data were mapped to hg38.9 using HISAT in the present study, which might cause the missing of important reads/genes and introducing more errors since HISAT is not robust enough. Third, the relationships between mRNAs and lncRNAs were yet to be confirmed. Furthermore, no experimental confirmation was performed for the identified biomarkers. Therefore, in the future work, with more RNA-Seq data and reliable mRNA-lncRNA interactions generated, more confident ICM susceptibility biomarkers could be identified, which would be further confirmed by other means, such as experiments.

## Conclusions

In summary, combining ceRNA triplets and functional information, eight ICM susceptibility mRNAs and eight ICM susceptibility lncRNAs identified by our susceptibility biomarker identification strategy could be susceptibility biomarkers and potential therapeutic targets for ICM. Given related ICM disease genes are rarely reported and stored in public disease associated databases at present, the ICM susceptibility biomarkers identified in the present study will contribute to the diagnosis and treatment of ICM. The proposed strategy based on significantly differentially expressed ceRNA triplets would contribute to other complex diseases without diseases biomarkers in public databases.

## Abbreviations

ceRNA: competing endogenous RNA
COPD: chronic obstructive pulmonary disease
ICM: ischemic cardiomyopathy
SVM: support vector machine
